# Noninvasive and quantitative intracranial pressure estimation using ultrasonographic measurement of optic nerve sheath diameter

**DOI:** 10.1038/srep42063

**Published:** 2017-02-07

**Authors:** Li-juan Wang, Yan Yao, Liang-shu Feng, Yu-zhi Wang, Nan-nan Zheng, Jia-chun Feng, Ying-qi Xing

**Affiliations:** 1 Department of Neurology, The Neuroscience Center, The First Hospital of Jilin University, Jilin University, Changchun, China; 2 Department of Epidemiology and Biostatistics, School of Public Health, Jilin University, Changchun, China

## Abstract

We aimed to quantitatively assess intracranial pressure (ICP) using optic nerve sheath diameter (ONSD) measurements. We recruited 316 neurology patients in whom ultrasonographic ONSD was measured before lumbar puncture. They were randomly divided into a modeling and a test group at a ratio of 7:3. In the modeling group, we conducted univariate and multivariate analyses to assess associations between ICP and ONSD, age, sex, BMI, mean arterial blood pressure, diastolic blood pressure. We derived the mathematical function “Xing & Wang” from the modelling group to predict ICP and evaluated the function in the test group. In the modeling group, ICP was strongly correlated with ONSD (r = 0.758*, p* < 0.001), and this association was independent of other factors. The mathematical function was ICP = −111.92 + 77.36 × ONSD (Durbin-Watson value = 1.94). In the test group, a significant correlation was found between the observed and predicted ICP (r = 0.76, *p* < 0.001). Bland-Altman analysis yielded a mean difference between measurements of −0.07 ± 41.55 mmH_2_O. The intraclass correlation coefficient and its 95%CIs for noninvasive ICP assessments using our prediction model was 0.86 (0.79–0.90). Ultrasonographic ONSD measurements provide a potential noninvasive method to quantify ICP that can be conducted at the bedside.

Elevated intracranial pressure (ICP) is a common emergency condition with poor clinical outcomes and high rates of mortality^1^,^2^. Evaluation of ICP is critical for the diagnosis of neurologic diseases. Direct methods, such as lumbar puncture (LP) and intraventricular catheterization, are currently used for ICP evaluation. However, the invasiveness of such procedures can result in complications, including haemorrhage and bacterial colonization. Furthermore, invasive ICP monitoring is not routinely undertaken due to the absence of neurosurgeons, and contraindications such as thrombocythemia or coagulopathy^3^. Because of these drawbacks, accurate noninvasive methods to measure ICP have long been sought. Early detection of increased ICP is incredibly important, and simple, reproducible noninvasive methods are urgently needed. Several methods have been proposed for noninvasive measurement of ICP, including magnetic resonance imaging (MRI), computed tomography (CT), transcranial doppler, and electroencephalography. However, these techniques have not proved accurate enough to be widely used in clinical diagnosis.

The optic nerve forms from an outgrowth of the diencephalon during embryogenesis. It is surrounded by the intraorbital subarachnoid space and both experience the same pressure changes^4^,^5^. A linear relationship has been found between ICP and optic nerve sheath diameter (ONSD)^6^. Furthermore, ultrasound measurements of the ONSD correlate closely with postmortem and *in vivo* MRI measurements^7^,^8^,^9^. Ultrasonic ONSD measurement takes about 5 minutes to complete at bedside, is easy to conduct, and is highly reproducible^10^, showing low intra- and inter-observer variation^11^. Therefore, ONSD measurement has been increasingly investigated as a tool for rapidly assessing the risk of increased ICP in several studies in Western countries^12^,^13^,^14^,^15^,^16^,^17^,^18^,^19^,^20^,^21^,^22^. Furthermore, variations in ONSD have been used to evaluate the efficacy of osmotherapy for elevated ICP^23^.

In our previous study, we confirmed that ONSD correlates with increased ICP in Chinese patients^24^. Published meta-analyses have suggested cut-off values from 4.8 mm to 5.9 mm indicating increased ICP^22^,^25^. However, these existing threshold values only provide a qualitative indication of increased ICP, and cannot provide quantitative values for the degree of ICP. Additionally, an ONSD research group has identified the need to determine whether the diagnostic accuracy of ONSD ultrasonography varies according to patient characteristics such as age and weight, etc^26^. Indeed, few studies have used ultrasonographic ONSD to noninvasively and quantitatively assess ICP values, or the additional factors that may influence these. To address these gaps in the literature, we estimated ICP by calculating a mathematical function, and subsequently evaluated the accuracy of these measurements in a new test population.

## Methods

### Study Population

This study was conducted in the First Hospital of Jilin University, which is a general public hospital in China that includes one of four ultrasound training centres in China. The study protocol was approved by the ethics committee of The First Hospital of Jilin University and all methods were performed in accordance with the relevant guidelines and regulations. All participants provided written informed consent.

This was a blind cross-sectional study that recruited patients suspected of having increased ICP for various reasons and who underwent LP between March 2014 and March 2015. Patients were excluded if they: (1) were aged < 18 or > 80 years old; (2) presented with ophthalmic diseases, such as inflammation, tumours, or traumas; (3) had a history of glaucoma or current medications that might affect cerebrospinal fluid (CSF) pressure, such as diuretics, carbonic anhydrase inhibitors, and glucocorticoids; or (4) had an ONSD boundary that contained artefacts or was otherwise unclear.

**Figure 1:**
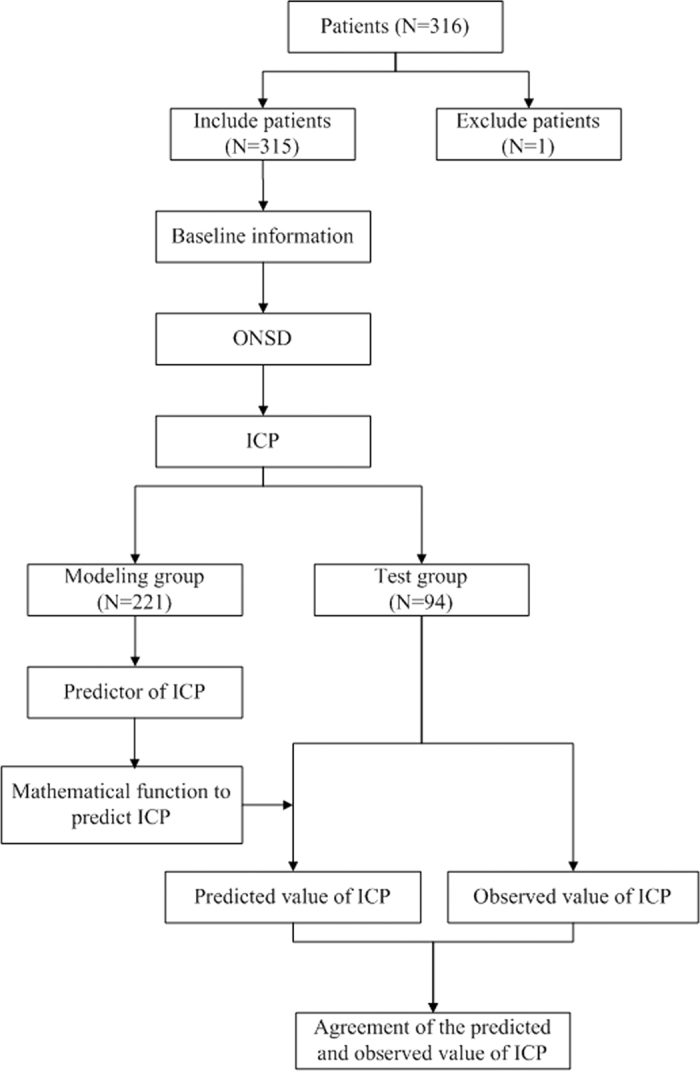
Flow diagram of the study. The terms “*n*” in the boxes represent the number of corresponding studies. ONSD = Optic nerve sheath diameter, ICP = intracranial pressure.

The following patient data were recorded: age, sex, waist circumference, head circumference, body mass index (BMI), systolic blood pressure (SBP), and diastolic blood pressure (DBP). Mean arterial blood pressure (MABP) was calculated as 1/3 × SBP + 2/3 × DBP. A computer randomly assigned patients to a modeling group and a test group at a ratio of 7:3^27^. In the modeling group, we investigated associations between ICP and patient parameters. We constructed a mathematical function named “Xing & Wang” to predict ICP from the statistically significant parameters found to be independently associated with ICP. Subsequently, we calculated predicted ICP values in the test group using the derived mathematical function. We then assessed agreement between the observed and predicted ICP values (Fig. 1).

### Measurements

Ultrasound examinations of the eyes were performed in B-mode on a Philips iU22 ultrasound system (Andover, Massachusetts, USA), using a 9–3 MHz linear array transducer. The acoustic output of the ultrasound system was adjusted to the requirements of orbital sonography according to the ALARA (“as low as reasonable achievable”) principle to avoid damage to the retina and lens^21^. The patients were examined in a supine position.

**Figure 2:**
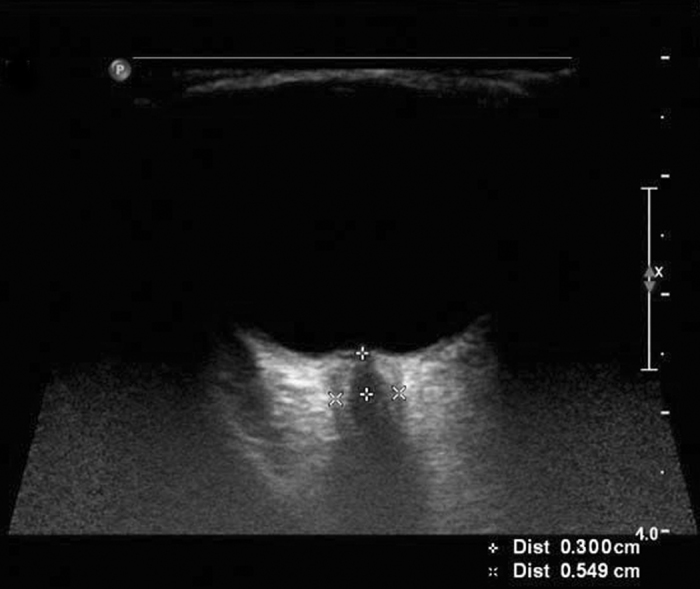
The measurement of optic nerve sheath diameter (ONSD). ONSD measurement was assessed 3 mm behind the orbit. The ONSDs of patients with elevated intracranial pressures (ICPs) were significantly enlarged.

Investigations of the ONSD of both eyes in all patients were independently performed by two experienced observers who were blinded to each other’s assessments and the state of illness of the patients. In our previous study, the ultrasonographic ONSD measurements were obtained by two observers and there were no significant differences between their measurements. Consistent with previous protocols, the probe was placed lightly over the closed upper eyelid with a thick ultrasound gel to prevent pressure being exerted on the eye. The position of the probe was adjusted to clearly display the entry of the optic nerve into the eyeball. We randomized the order in which the left and right eyes were measured. The first measurement was performed in the sagittal plane (with the probe in a vertical orientation), and the second measurement was performed in the transverse plane (with the probe in a horizontal orientation)^14^,^28^. ONSD was assessed bilaterally, 3 mm behind the globe^29^,^30^ (Fig. 2). Each observer performed the measurements twice, such that each ONSD was measured eight times in total. To minimize variability, the final ONSD value for both eyes of each patient was derived from the average of the 16 values from the two observers.

After the ONSD measurements, LP was immediately performed by an experienced neurological resident who was blind to the ultrasonographic ONSD results. The opening pressure of the CSF was recorded in mm of water pressure (mmH_2_O) using LP. Patients were awake and placed in the left lateral position with their hips and knees flexed and their heads as close to their knees as comfortably possible. The area around the lower back was prepared using an aseptic technique. The patient was asked to relax. Once the subarachnoid space had been entered, the patient was asked to straighten his or her legs, after which the opening pressure on the LP was recorded and the fluid samples were obtained. Elevated ICP was defined as a pressure > 200 mmH_2_O^31^,^32^.

### Statistical Analysis

Statistical analysis was performed using a commercial statistical software package (SPSS for Windows, version 17.0; IBM-SPSS, Chicago, IL, USA). Continuous variables are reported as the mean ± the standard deviation (SD), and categorical variables as the frequency and percentage.

The distribution of values was assessed using the Kolmogorov-Smirnov test. We assessed differences in demographic variables, ONSD, and ICP between the modeling group and the test group using two-tailed Student’s t-tests. A χ^2^ test was used to compare proportions. All P-values were two-tailed.

In the first step of the statistical analysis, we submitted data from the modeling group to univariate analyses to identify associations between ICP, BMI, MABP, DBP, age, sex, head circumference, waist circumference, and ONSD. In the second step, multiple linear regression models were constructed to identify parameters that were significantly and independently associated with ICP. This generated an equation linking ICP and ONSD in the modeling group data. The presence of serial correlations among the residuals was tested using the Durbin-Watson statistic. A Durbin-Watson statistic between 1.5 and 2.5 indicated that no serious residual autocorrelation was present. In the third step of the analysis, we tested the equation predicting ICP in the test group data. Bland-Altman analysis was applied to evaluate the accuracy and precision of the prediction. We calculated intraclass correlation coefficients (ICC) and 95% confidence intervals (CIs) for the comparison of observed and predicted values of ICP to determine the prediction’s reliability.

## Results

In total, 316 Chinese patients were recruited. One subject with missing data was excluded. No patients had an ONSD boundary that contained artefacts or was otherwise unclear in our study, such that 315 participants (mean age, 41.03 ± 15.00 years; range, 18 to 80 years; 174 males; 122 patients with elevated ICP) were included in the study. The mean ONSD for all participants was 3.95 ± 0.67 mm (median, 3.78 mm; range, 2.52 to 6.22 mm). The indications for LP were cerebral infections, cerebrovascular disease, hydrocephalus, primary headache, infarcts, epilepsy, peripheral neuropathy, and cranial nerve palsy. The subjects were randomly assigned by a computer to a modeling group and a test group at a ratio of 7:3, resulting in 221 patients assigned to the modeling group and 94 assigned to the test group. Demographic data for the modeling and test groups are presented in Table 1. Due to the random assignment of participants to the modeling or test group, these did not differ significantly in age, gender, BMI, waist circumference, head circumference or arterial blood pressure (all *p* > 0.05).

**Figure 3:**
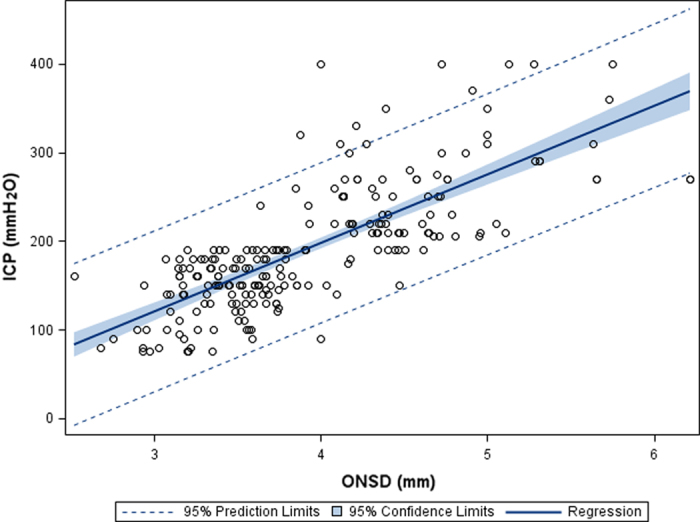


In the modeling group *(n* = 221), ICP was strongly correlated with ONSD (Pearson correlation: r = 0.758*, p* < 0.001; Fig. 3). Additionally, the ICP values were significantly associated with age (r = −0.255, *p* < 0.001), waist circumference (r = 0.135, *p* = 0.017), and BMI (r = 0.177, *p* = 0.002). In our study population, ICP was not significantly associated with head circumference (r = 0.05, *p* = 0.375), MABP (r = 0.013, *p* = 0.812), or DBP (r = 0.046, *p* = 0.495). A multivariate linear regression analysis was performed to select the variables that are independently associated with ICP, including age, gender, BMI, waist circumference, head circumference, and DBP. Only ONSD remained in the final model (Table 2).

**Figure 4:**
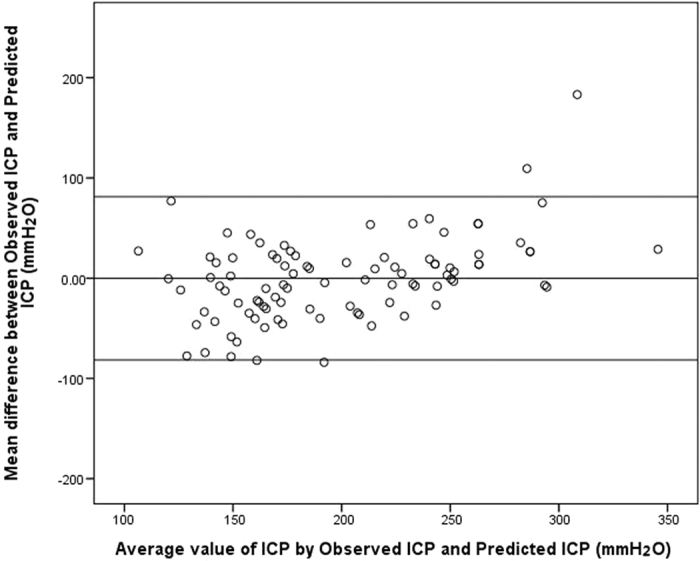


In the modeling group, we used the ONSD to built the mathematical function “Xing & Wang” to predict ICP. Predicted ICP = −111.92 + 77.36 × ONSD. The Durbin-Watson value of the function was 1.94. The mean residual value was 0 (t = 1*, p* = 1.0), and the residuals were normally distributed (Z = 0.81*, p* = 0.53). The results of the Bland-Altman analysis are shown in Fig. 4.

In the test group (*n* = 94), a significant correlation was found between the observed and predicted ICP (r = 0.76, *p* < 0.001). Bland-Altman analysis yielded a mean difference between measurements of −0.07 ± 41.55 mmH_2_O. The mean of the difference plus or minus the 1.96-fold standard deviation of the difference (mean ± 1.96 SD) was 81.37 and −81.52 mmH_2_O, respectively. The ICC (95%CIs) for the noninvasive ICP assessment using the prediction model was 0.86 (0.79–0.90).

## Discussion

Our data showed that ONSD was independently associated with ICP after removing variance from other factors. We also successfully derived a mathematical function to quantitatively assess ICP, which was highly consistent with observed values. Thus, our results indicate that ultrasonographic measurement of ONSD could be a strong quantitative predictor of elevated ICP.

In 2013, Dubourg *et al*. indicated a need to determine whether the diagnostic accuracy of ONSD ultrasonography varies according to patient characteristics^26^. We therefore investigated such factors in the current study. Multivariate linear regression analysis showed that ONSD was a significant, independent predictor of ICP. Sex, age, BMI, waist circumference, head circumference, and DBP did not influence the relationship between ONSD and ICP. As such, ONSD values were used to successfully estimate ICP.

Although ultrasound measurement of ONSD is increasingly used as a marker to detect elevated ICP, there is no consensus regarding a definitive threshold for elevated ICP^22^,^25^. Most studies have suggested a cut-off point of 5 mm^19^,^31^,^33^. However, such a threshold can only qualitatively diagnose increased ICP, but does not provide quantitative values for ICP. Elevated ICP can increase the likelihood of poor clinical outcomes and high mortality rates. Therefore, having a quantitative value for ICP would prove more useful in determining disease severity and prognosis. To the best of our knowledge, no previous study has quantified ICP noninvasively using ultrasonography. We therefore derived a mathematical function to provide such quantification. We calculated an ICC (95%CIs) for noninvasive ICP assessment using the prediction model of 0.86 (0.79–0.90), which is indicative of strong agreement between the observed and estimated ICP values. Therefore, we propose that ultrasonographic measurement of ONSD provides a potential tool for quantitative and noninvasive evaluation of ICP.

Very few reports have provided noninvasive quantification of ICP. Xie *et al*. proposed, and subsequently confirmed clinically, that ICP could be estimated quantitatively based on MRI-assisted orbital subarachnoid space width measurements^27^. However, their study included 72 patients, only eight of which had elevated ICP. Therefore, the authors suggested their results could primarily be applied to patients without elevated ICP. The results reported here show that ICP can be quantitatively and noninvasively evaluated using ultrasonographic measurement of ONSD in a larger sample of 315 subjects, 122 of whom had elevated ICP. Additionally, all ICP values in the previous study were less than 26.5 mmHg^27^, whereas some patients in our sample had markedly elevated values of up to 30.1 mmHg (400 mmH_2_O). We believe the larger sample and the greater range of ICP values greatly increase the clinical applicability of our proposed technique.

Chen reported that the median ONSD value in healthy adults was 5.1 mm and the 95^th^ percentile was 5.9 mm^34^. However, there are several differences between Chen’s study and our study. Firstly, the participants in Chen’s study were healthy, while ours had normal or elevated ICP. Secondly, Chen measured ONSD twice in the horizontal plane and calculated the mean value. We measured sagittal and horizontal sections twice, and the final ONSD measurement value for each patient was derived from the average of 16 values from both eyes assessed by two observers to minimize variability. Other previous studies have investigated ONSD in healthy people. Goeres *et al*. reported a mean ONSD of 3.68 mm (95% confidence interval [CI], 2.85–4.40) in healthy Canadians^35^. Soldatos *et al*. reported a median ONSD of 3.6 ± 0.6 in healthy Greek participants^36^. Ballantyne *et al*. found an average ONSD of 3.4–3.6 mm in healthy British people^11^. Therefore, there is no clear consensus regarding the median ONSD in healthy adults. Furthermore, the reported cut-off value for elevated ICP has varied from 4.6–5 mm^12^,^19^,^31^,^33^. As such, diagnostic criteria for elevated ICP-based ultrasonographic ONSD have not been established.

Our study does have some limitations. First, the model may underestimate the true ICP value in patients with extremely high ICP. As such, the elastic modulus of the optic nerve sheath at higher ICPs should be investigated in future research. Second, a more accurate mathematical function would be useful to enable a wider application of ultrasonographic ONSD measurements to quantitatively evaluate patients at risk of elevated ICP, as well as increasing the reliability of the technique. The Bland-Altman analysis in this study suggested that any estimate might be deviate by as much as ±80 mmH_2_O. To increase the clinical applicability, large-scale, multi-centre clinical trials are required in the future. Third, although ultrasonographic ONSD measurement was assessed 3 mm behind the orbit, different measurement depths for measuring ONSD have been investigated in CT and MRI studies^27^,^37^. Accordingly, future studies should identify the optimal site for measuring ONSD by ultrasonography. Despite these limitations, our study indicates that noninvasive measurements of ONSD could be used to quantitatively evaluate ICP, and we hope our results will inform and encourage further research to refine the model.

## Conclusion

The measurement of ONSD using ultrasonography provides a highly practical method to assess ICP. Being noninvasive, ONSD ultrasonography provides a potential tool for rapid quantification of ICP, even at the bedside. This technique, especially when coupled with an accurate mathematical formula for predictive use, may be helpful for screening patients with elevated ICP, especially when invasive ICP monitoring is contraindicated.

## Additional Information

**How to cite this article**: Wang, L.-j. *et al*. Noninvasive and quantitative intracranial pressure estimation using ultrasonographic measurement of optic nerve sheath diameter. *Sci. Rep.* **7**, 42063; doi: 10.1038/srep42063 (2017).

**Publisher's note:** Springer Nature remains neutral with regard to jurisdictional claims in published maps and institutional affiliations.

## Figures and Tables

**Table 1:** Demographic data for the modelling and test groups.

	**Modelling group (n = 221)**	**Test group (n = 94)**
Male (n, %)	122 (55.20%)	52 (55.32%)
Age (years)	40.39 ± 14.73	42.53 ± 15.56
ONSD (mm)	3.914 ± 0.69	4.038 ± 0.62
BMI (kg/m^2^)	23.98 ± 3.51	24.07 ± 4.53
Waistline (cm)	78.87 ± 9.73	81.59 ± 11.95
Head circumference (cm)	55.09 ± 2.15	55.64 ± 1.89
MABP (mmHg)	94.39 ± 12.11	96.81 ± 14.78
DBP (mmHg)	79.52 ± 10.93	81.57 ± 12.75
ICP (mmH_2_O)	190.88 ± 70.05	200.37 ± 58.87

ONSD = optic nerve sheath diameter, BMI = body mass index, MABP = mean blood pressure, DBP = diastolic blood pressure, ICP = intracranial pressure.

**Table 2:** Multivariate linear regression analysis of variables associated with ICP (*n* = 221).

**Variable**	**beta**	**SE**	**P**
ONSD	75.21	4.82	< 0.001
Age	−0.42	0.23	0.067
Sex	−11.66	6.85	0.090
BMI	0.384	1.44	0.790
Waistline	0.502	0.56	0.370
Head circumference	−0.89	1.62	0.582
DBP	0.324	0.29	0.271

ONSD = optic nerve sheath diameter, BMI = body mass index, DBP* *= diastolic blood pressure. For the regression model, *p* < 0.0001 and R^2^ = 0.59.
